# Visualisation ergonomics and robotic surgery

**DOI:** 10.1007/s11701-023-01618-7

**Published:** 2023-05-19

**Authors:** Shing Wai Wong, Philip Crowe

**Affiliations:** 1 Department of General Surgery, Prince of Wales Hospital, Sydney, NSW Australia; 2 Randwick Campus, School of Clinical Medicine, The University of New South Wales, Sydney, NSW Australia

**Keywords:** Visualisation, Ergonomics, Robotic surgery, Visual fatigue

## Abstract

Stereopsis may be an advantage of robotic surgery. Perceived robotic ergonomic advantages in visualisation include better exposure, three-dimensional vision, surgeon camera control, and line of sight screen location. Other ergonomic factors relating to visualisation include stereo-acuity, vergence–accommodation mismatch, visual–perception mismatch, visual–vestibular mismatch, visuospatial ability, visual fatigue, and visual feedback to compensate for lack of haptic feedback. Visual fatigue symptoms may be related to dry eye or accommodative/binocular vision stress. Digital eye strain can be measured by questionnaires and objective tests. Management options include treatment of dry eye, correction of refractive error, and management of accommodation and vergence anomalies. Experienced robotic surgeons can use visual cues like tissue deformation and surgical tool information as surrogates for haptic feedback.

## Introduction

Visualisation is one of the two facets of sensory ergonomics in surgery. There are important ergonomic factors to consider with regards to visualisation in robotic surgery. Most are advantageous, but some are detrimental [1]. Conventional laparoscopic surgery has several drawbacks that can impair complete visualisation of the operative field, which is presented as a monocular view on a two-dimensional (2D) video display. The surgeon relies on an assistant to control the camera and must contend with the loss of peripheral vision and potential inadvertent tissue injury. The surgeon also loses depth perception and instead relies on visual cues, such as light and shade, relative size, and motion parallax to give a sense of depth [2]. However, indirect depth cues can be degraded in situations of limited light such as operating in the pelvis or in situations of limited contrast such as within dark blood-stained tissue [3]. In addition, the disparity between the location of the display monitor and the actual operative field (where we look versus where we operate) can result in visual and mental fatigue.

Robotic surgery ameliorates these issues by providing better exposure, immersive three-dimensional (3D) vision, surgeon camera control, and line of sight screen location. There are other visualisation ergonomic factors, some of which may be detrimental, to consider when assessing the difference between laparoscopic and robotic systems. This includes stereo-acuity, vergence–accommodation mismatch, visual–perception mismatch, visual–vestibular mismatch, visuospatial ability, visual fatigue, and visual feedback to compensate for lack of haptic feedback. Some of the detrimental visualisation ergonomic factors can be overcome with experience and adaptation by the brain sensory system.

## Exposure

Visualisation exposure can be enhanced with robotic surgery because of better illumination, magnification, and better viewing angles. Optimal surgical illumination should offer sufficient ambient light with the ability to focus on the surgeon’s operative field even at difficult angles; without shadow, glare, and artifact [4]. Light emanating from the laparoscope provides more focussed illumination compared with overhead lights during open surgery. Degradation of the light cable used in laparoscopic surgery can reduce the intensity of light. Improved visualisation can occur using the robot camera magnification technology to position the camera further away from a smoke or steam generating dissection site (e.g., during pelvic surgery). Use of magnification together with motion scaling can improve vision and accuracy [5]. The port hopping feature of the *da Vinci Xi* robot (currently the most recognised and widely used robot-assisted system) allows re-positioning of the camera to facilitate optimal multi-quadrant visualisation during surgery [6].

## Three-dimensional vision

3D visualisation provided by the robotic console can reduce ocular strain, task performance time, and error rates [4] Falk et al. found that peak velocity was reduced (p = 0.01) and the deceleration phase of motion was prolonged (*P* < 0.05) in a study of 15 surgeons performing six tasks with 2D compared with 3D vision [7]. 3D laparoscopy has been shown to shorten operative times and improve precision when compared to 2D laparoscopy [8]. A meta-analysis comparing 3D laparoscopic system with 2D laparoscopy in the treatment of different urological conditions found shorter operative time (*p* < 0.0001) and lower estimated blood loss (*p* = 0.001) for radical prostatectomy surgery [9]. However, 3D vision has not been shown to be superior to two-dimensional vision for all laparoscopic surgery [9, 10]. 

The modern robotic console may provide a better stereopsis experience for surgeons compared to the traditional 3D images created by shuttering or polarizing images [11]. The *da Vinci* robotic system uses two separate optical trains with two independent three-chip cameras and display systems to present slightly different images to each eye, mimicking binocular vision. The optical system minimises geometric distortion across the field of view, enabling stereo fusion of images even near the edges.

## Surgeon camera control

Robotic surgery returns the control of the camera to the operating surgeon on a stable platform, resulting in a steadier view of the operative field with better centering, horizon adjustment, zoom control, and instrument visualisation. In a simulation study of 84 medical students, surgeon performance was significantly influenced by the quality of the laparoscopic camera navigator [12]. The use of robotic laparoscopic holder has been shown to provide consistent control of the view of the operative field [13]. The quality of camera navigation has been shown to be better with robotic versus conventional laparoscopic rectal cancer surgery [14]. 

## Line of sight screen location

The *da Vinci* console returns the screen location back in line with the hands of the surgeon, as occurs naturally in open surgery. This view of the surgeon’s hands performing a task is intuitive and familiar. Studies looking at the optimal position of image display in endoscopic surgery found that task performance improves when the image display is close to the hands [15]. Hanna et al. found endoscopic knot tying was better (*P* < 0.01) and faster (*p* < 0.01) with hand-level “gaze-down” viewing than eye-level viewing in a laboratory study of ten endoscopic surgeons [10]. In addition, the downgaze viewing results in a smaller ocular surface for exposure to the effects of tear film evaporation compared with the horizontal gaze when viewing the screen during laparoscopic surgery. [16]

## Stereo-acuity

Stereopsis is a binocular sensation of relative depth caused by horizontal disparity of retinal images from both eyes which are then cortically integrated [17]. Stereo-acuity is a measure of the smallest depth that an individual can detect with binocular disparity [3]. Stereo-acuity is variable among individuals and declines with age [18]. In a study of 66 surgeons, reduced or no stereopsis was found in 2–14% depending on the test used [19]. Worse stereopsis with age might be due to insufficient correction of refractive error [20]. Hence, the visualisation advantage of 3D robotic surgery over the conventional 2D surgery may not be significant for some surgeons.

Stereopsis can be measured globally by random dot stereo tests and locally by contour stereo tests [18]. Images of stereo figures are embedded in a background of random points with random dot stereo tests and testing requires wearing anaglyph red and green glasses. Images are separated and presented separately to each eye with contour stereo tests and testing requires wearing cross-polarizing filtered glasses. Contour stereo tests have the disadvantage of monocular cues. High-quality stereo vision of the surgeon has been shown to offer no advantage when using the 2D display but associated with significantly reduced operating time and error when using the 3D display [18]. Gietzelt et al. found that surgical experience could partly compensate for a poorer stereo-acuity [18]. 

## Vergence–accommodation mismatch

With normal visual activity and 2D laparoscopy, the eyes automatically accommodate and converge to the same distance on a specific object or on the display screen [3]. Vergence and accommodation are neurally coupled, with accommodative changes evoking changes in vergence (accommodative vergence), and vergence changes evoking changes in accommodation (vergence accommodation) [21]. Accommodative vergence can be detected only during monocular fixation, because the need for binocular fusion is the stimulus to convergence. Fincham and Walton confirmed the evidence of vergence accommodation by measuring accommodation both during condition of constant light vergence and changed convergence, as well as condition of constant convergence and changed light vergence [21]. Vergence–accommodation mismatch (VAM) can occur with 3D vision associated with closed robotic console systems, because the accommodation distance is fixed from the surgeon’s eyes to the display screen and the vergence distance depends on the simulated distance.

This VAM may be influenced in different ways with laparoscopic and robotic 3D vision [10, 22, 23]. The changes in convergence and accommodation (and mismatch) at the typical viewing distance of 50 cm with laparoscopy and open robotic console systems are much smaller than the head-mounted displays of closed robotic console systems. On the other hand, in contrast with robotic surgery, rapid movement of the camera in laparoscopic surgery exaggerates 3D display vergence–accommodation mismatch [24]. 

To see objects clearly on 3D displays requires the viewer to counteract the neural coupling between vergence and accommodation. Visual discomfort and fatigue can occur because of this vergence–accommodation conflict. Optimising focus cues (accommodation and blur in the retinal image) can improve stereo-acuity, reduce distortions, and reduce viewer fatigue and discomfort [25]. 

## Visual–perception mismatch

Visual perception is related to how visual information is processed by the brain. Perceptual learning can lead to performance improvement through practice or training [26]. Visual–perception mismatch (VPM) occurs with laparoscopic and robotic surgery but can be reduced with increasing experience. With laparoscopic surgery, the fulcrum point created by the trocar at the abdominal wall results in counterintuitive movements of the hand and the laparoscopic instrument, creating a visualisation–perception disconnect [27]. The surgeon console–robot interface of robotic systems removes the fulcrum effect through software technology. The robotic system can translate the surgeon’s movements exactly resulting in surgeon hand movements replicated by movement of the instrument tip in the same direction. However, the technological advances of motion scaling and filtering of surgeon tremor available on the robotic system can lead to a discrepancy between vision and surgeon hand movements.

With the robotic system, the use of the clutch control (which allows greater movement of the robotic arms from the smaller confines of the surgeon console) can potentially introduce a visual–perceptual mismatch that can add an additional cognitive load [28]. Abiri et al. found significantly more peg drops (*P* = 0.011) and longer time to completion with a peg transfer task (*P* < 0.001) in 45 novice surgeons operating with a single-axis misalignment [28]. With robotic surgery, use of the clutch can lead to a brain sensory misalignment of the location of surgeon hands at the console and the location of the robotic arms as visualised through the console binoculars. However, preference by experienced surgeons to maintain their hands in an ergonomic neutral position may allow them to unconsciously overcome this VPM.

## Visual–vestibular mismatch

Visual–vestibular mismatch (VVM) can occur when a person is overusing their visual cues for determining their position in space, when it might be more appropriate to use the information from the inner ear vestibular organs (which sense orientation, movement, and balance). This can occur during laparoscopic surgery if the assistant is not keeping the laparoscope steady and the surgeon may interpret visual motion as self-motion. With robotic surgery, the camera is held more steadily and VVM may be less of a problem.

Visual and vestibular signals are the primary sources of sensory information for self-motion. Conflict among these signals is adaptable but can result in vertigo, postural instability, and simulator sickness [29]. There is a trade-off between visual–vestibular integration and conflict detection that is mediated by eye movements [30]. Compared with laparoscopic surgery, closed robotic console surgery results in less head movement with resting of the forehead on the head rest and the eyes on the binoculars. This results in more eye movements and less retinal motion when the visual target is redirected. Therefore, use of the robotic visualisation system can improve conflict detection at the cost of impaired integration performance. [30]

## Visuospatial ability

Visuospatial ability (VSA) is correlated with surgical skill, especially in less-experienced surgeons at the beginning of their learning curve. VSA may be somewhat innate but can often be improved with training. VSA can be tested with the Perceptual Ability Test (PAT) which tests a person’s ability to interpret 2D shapes in a 3D context. The PAT has been validated and shown to correlate with technical performance [31]. Better VSA has been shown to correlate with improved performance by novice surgeons on a robotic surgery simulator [32]. High PAT performers had a higher simulation score (49% higher, *p* = 0.005) and completed the exercise faster (36% faster, *p* = 0.009) than the low PAT performers. Higher initial VSA may result in greater improvement in performance and time with training especially for complex tasks [33]. 

## Visual fatigue

Longer robotic operating times also put the surgeon at risk of visual fatigue from constant viewing of the video display through the binoculars, leading to computer vision syndrome, which includes symptoms such as blurred vision, dry eyes, eyestrain (asthenopia), and headache [34, 35]. There are two main causes of visual fatigue: external symptoms relating to dry eye and internal symptoms relating to accommodative or binocular vision stress [36]. These symptoms have been previously studied in office workers and computer gamers. A study found that more than 4 h of computer gaming was associated with both ocular and physical discomfort [35]. In a survey of 432 robotic surgeon, eye symptoms were found to be more common with longer years practicing robotic surgery [23]. The causes of visual discomfort when viewing stereo displays include cross-talk between the two eye images, misalignment of the images, head position, vergence–accommodation mismatch, visibility of flicker or motion artifacts, and visual–vestibular conflicts [24, 37]. 

Digital eye strain may be measured subjectively with questionnaires or objectively with critical flicker-fusion frequency, blinking and squinting characteristics, accommodative function, and pupil characteristics [36, 38, 39]. Management approaches include correction of refractive error, management of dry eye (downgaze, lubricating eye drops, omega-3 fatty acids supplementation, and blink efficiency training), regular screen breaks (20/20/20 rule of 20 s break every 20 min to view a distant object at 20 feet), consideration of vergence and accommodation problems, and use of blue-light filtering lenses [36]. 

## Visual feedback

Experienced robotic surgeons can use visual cues like tissue deformation as surrogates for force and haptic feedback (which is not available with the *da Vinci* robotic system). Meccariello et al. performed a study on 25 surgeons assessing their ability to recognise the thickness of custom-made membranes without use of haptic feedback [40]. They found a significant difference in performance/average score between experts and non-experts (*p* < 0.05). In addition, 67% of expert surgeons correctly identified the location of a metal clip in the correct quadrant of a membrane compared to 37% of non-experts. Restoration of three-dimensional vision may be important to compensate for loss of force feedback, with experienced surgeons able to feel a pseudo-tactile sensation [41, 42]. Vision-based approaches to predict force can also use surgical tool information, such as the tool-tip trajectory, velocity, and grasper status [43]. Combining information arising from different sensory modalities is essential to interact effectively with the environment. Cross-modal interactions between different sensory modalities may be automatic and unconscious. Lunghi et al. used continuous flash suppression to investigate whether haptic signals can interact with visual signals outside of visual awareness. They found that touch can accelerate the rise to consciousness of a suppressed visual stimulus but must be congruent, with matching in either spatial frequency or orientation [44]. The perception of haptic feedback may be explained by the converse relationship of this visual–haptic congruency.

## Open robotic console systems

New robotic surgical systems have been developed and are now available for use since the expiration of patents held by Intuitive Surgical (makers of the *da Vinci* robotic system). The Cambridge Medical Robotics Versius, the Medtronic Hugo, and the TransEnterux Surgical Senhance robotic systems all provide an open console system which require the use of 3D polarising glasses [45–47]. The open console design enables better verbal and nonverbal communication between the surgical team members. The visualisation ergonomic considerations for the closed *da Vinci* system would differ for the open console systems but has not been compared directly. The visualisation advantages of the open console system include improved camera control, less reliance on visual cues for haptic feedback, and potentially less VAM and VVM. The Senhance surgical system offers the added advantage of camera control by eye movements through an infrared eye-tracking system [45]. Both the Versius and Senhance robotic systems offer haptic feedback from the handles. The visualisation disadvantages of the open console system relate to the need to use polarised glasses like for 3D laparoscopic surgery, and include decrease brightness of the operating field, need for horizontal gaze, less stereopsis, and more visual fatigue [45]. In addition, visualisation with the Hugo system is complicated by its two monitors: a 3D-HD screen with head tracking system and a right sided touchscreen surgeon interactive display which can select visual filters and rotate the camera [48]. 

## Conclusion

Understanding the ergonomics of visualisation during robotic surgery is important. Stereopsis requires visual cortex fusion of two slightly different images from each of the eyes. Optimal visualisation occurs when VAM and cross-modality conflict relating to the sensations of proprioception and balance are compensated. Visual fatigue from viewing stereoscopic images is more common when the images are outside the depth of focus and can be caused by cross-talk, misalignment, head position, VAM, and VVM. Management approaches include correction of refractive error, use of lubricating eye drops, blink efficiency training, and regular screen breaks.

## Data Availability

Not applicable.
